# The Role of C-Reactive Protein and the SOFA Score as Parameter for Clinical Decision Making in Surgical Patients during the Intensive Care Unit Course

**DOI:** 10.1371/journal.pone.0055964

**Published:** 2013-02-07

**Authors:** Zainna C. Meyer, Jennifer M. J. Schreinemakers, Paul G. H. Mulder, Ruud A. L. de Waal, Antonius A. M. Ermens, Lijckle van der Laan

**Affiliations:** 1 Department of Surgery, Amphia Hospital, Breda, The Netherlands; 2 Amphia Academy, Amphia Hospital, Breda, The Netherlands; 3 Department of Intensive Care Medicine, Amphia Hospital, Breda, The Netherlands; 4 Laboratory for Clinical Chemistry and Hematology, Amphia Hospital, Breda, The Netherlands; D’or Institute of Research and Education, Brazil

## Abstract

**Introduction:**

C-reactive Protein (CRP) is used next to clinical scoring systems to recognize critically ill patients prone to develop complications on the Intensive Care Unit (ICU). The purpose of this study is to assess the predictive value of CRP as parameter for clinical deterioration and/or clinical decision making as ordering diagnostic procedures or performing (re)interventions. Also, we wanted to determine the value of CRP in early detection of surgical complications in the critically ill general surgical patient in the ICU and its interpretation in adjunct to a clinical scoring system, the Sequential Organ Failure Assessment Score.

**Materials and Methods:**

In our prospective observational study, 174 general surgical patients admitted into the Intensive Care Unit were included. We evaluated the Sequential Organ Failure Assessment Score (SOFA) and daily measured the C-reactive protein (CRP) concentrations. All events (diagnostic or therapeutic interventions) and surgical complications were registered. Then the relationship between SOFA score, CRP concentrations, events and complications were studied.

**Results:**

Each 10% increase in CRP resulted in a 3.5% increase in the odds of an event (odds ratio 1.035, 95% CI: 1.004–1.068; *p* = 0.028). However, an increase in CRP levels did not lead to a higher odds of complication (OR 0.983, 95% CI: 0.932–1.036; *p* = 0.52). When adjusting for the SOFA score the effect of CRP on the probability of a first event remained significant (OR 1.033, 95% CI: 1.001–1.065; *p* = 0.046), and again did not significantly affect the complication probability (OR 0.980, 95% CI: 0.929–1.035; *p* = 0.46).

**Conclusions:**

An increase in C-reactive protein is a poor parameter for early detection of complications in the critically ill surgical patient in the ICU by means of diagnostic procedures or therapeutic (re)-interventions.

## Introduction

C-reactive protein (CRP) is an acute phase protein synthesized by the liver, which levels raise in response to inflammation. It is a sensitive but non-specific inflammatory biomarker often used as an indicator for systemic inflammatory response syndrome (SIRS) [1], [2] secondary to surgery or early postoperative complications [3]. However, its role as predictor for clinical deterioration in the surgical critically ill patient in the intensive care unit (ICU) remains unspecific.

Many classification systems have been developed to recognize early deterioration in critically ill patients. An important scoring system is the Sequential Organ Failure Assessment Score (SOFA score) which describe the clinical course of the patient as marker for the degree of organ dysfunction and predictor for mortality [4].

Next to the clinical scoring systems, other conventional markers are used to recognize these specific patients who are prone to develop complications, for example CRP. Studies about the value of CRP in the critically ill patient on the ICU are contradictory. Increased CRP concentrations in a heterogeneous population on the ICU have been associated with organ failure, prolonged ICU stay, high infection rates and mortality rates [3], [5], [6]. On the contrary, a recent review on the predictive value of CRP concentrations for survival concluded that CRP is not a good predictor for survival in the critically ill patient during the early course. Yet it may help to identify patients who are at risk for death [7].

Despite contradictory studies, trends in CRP concentrations during the ICU admission are frequently used to determine whether or not further, more invasive, diagnostic procedures and/or therapeutic interventions are required. An increase in CRP levels has been described as a crucial indicator for the diagnosis of postoperative complications in surgical patients such as infection, SIRS, sepsis, anastomotic leakage or mesenterial ischemia [8], [9], [10]. In addition, in patients with CRP levels >140 mg/L on the 4th postoperative day after rectal surgery with primary anastomosis, a 90.5% positive predictive value for postoperative infection was measured [3], [9], [11]. However, CRP concentrations change throughout the postoperative course in both subjects with or without complications, and they are not specific for any kind of complications [9]. The question remains if this parameter is correctly interpreted in clinical decision making. To our knowledge, there is very little information available on the predictive value of changes in CRP concentrations in the critically ill general surgical population in the ICU with regards to diagnostic procedures or (re)interventions.

The purpose of this study is to assess the predictive value of CRP concentrations as parameter for clinical deterioration and/or clinical decision making as ordering diagnostic and therapeutic (re)interventions in a heterogeneous group of surgical patient on the ICU. Furthermore, we wanted to determine the value of CRP in detection of surgical complication in the critically ill surgical patient in the ICU and its interpretation in adjunct to a clinical scoring system, the SOFA score.

## Materials and Methods

All surgical patients, admitted to the level three general surgical Intensive Care Unit at the Amphia hospital Breda, a tertiary health care institute, were prospectively included into the study when inclusion and exclusion criteria were fulfilled. Data were collected from April 2010 until June 2011. Inclusion was regardless of the indication for admission. The study was approved by the Institutional Review Board of the Amphia Academy Breda and the need for informed consent was waived.

Exclusion criteria were defined as non-surgical patients or cardiac surgery patients, duration of stay shorter than two days and patients younger than 18 years of age at time of admission into the ICU. The duration of follow-up was until the day of discharge or set at a maximum of 28 days. In case that more than one ICU-episode was observed within the same patient, only the first episode was chosen in order to meet the assumption of independence of observations.

Patient characteristics, initial surgery type and diagnosis for admission into the ICU were collected. Definition for systemic inflammatory response syndrome (SIRS) and sepsis, as described by Bone [1], [2], were used for the determination of severity of infection at admission. Also, the Acute Physiology and Chronic Health Evaluation II (APACHE II) as score for morbidity was calculated. Laboratory findings for C-reactive protein concentrations were also recorded. Diagnostic and therapeutic interventions and their outcome, surgical complications, length of stay and death were recorded as well.

All patients admitted into the ICU underwent standard care, including daily routine laboratory tests (with CRP concentrations) and a plain chest X-ray. Prophylaxis for ventilator-associated pneumonia was started when a duration of stay of 72 hour or longer was expected and/or mechanical ventilation for more than 48 hours was started (SDD). This prophylaxis included Cefotaxim 1 gram 4 times daily continued for four days and Orabase protective paste 4 times daily applied in the mouth and administered through a nasogastric tube.

### Sampling and Laboratory Analysis

Routine laboratory tests (hematology and biochemistry) were taken for all patients admitted into the ICU as part of the standard care. CRP concentrations were quantified using particle-enhanced immunologic turbimetric measurement for C-reactive protein Gen 3 in serum, as described by Roche (according to manufacturer’s instructions, Cobas 6000).

### Outcome Variables

The Sequential Organ Failure Assessment Score (SOFA score) was used as a marker for organ dysfunction over time in patients on the Intensive Care Unit. This simple scoring system daily assigns 1 to 4 points to each of the six organ systems examined depending on the level of dysfunction. The tracts used in this scoring system are pulmonary, cardiovascular, coagulation function, hepatic, renal and neurological (Table 1) [4].

**Table 1:** The Sequential Organ Failure Assessment Score.

SOFA score	1	2	3	4
*Respiration*	<400	<300	<200	<100
PaO_2_/FiO_2_mmHg			With respiratory support	With respiratory support
*Coagulation*	<150	<100	<50	<20
Platelets x 10^3^/mm^3^				
*Liver*	20–32	33–100	101–203	>203
Bilirubin µmol/L				
*Cardiovascular*	MAP	Dopamine ≤5	Dopamine >5 or	Dopamine >15
Hypotension^1^	<70 mmHg	or Dobutamine (any dose)	epinephrine ≤0.1 or norepinephrine ≤0.1	or epinephrine >0.1 or norepinephrine >0.1
*Central Nervous System*	13–14	10–12	6–9	<6
Glasgow Coma Score				
*Renal*	110–170	171–299	300–440 or <	>440 or <
Creatinine, µmol/L or urine output			500 mL/day	200 mL/day

To define the SOFA score, biochemistry data and clinical parameters of patients were collected at 5 o’clock a.m. during routine controls on the Intensive care unit.

1 Adrenergic agents administered at least in hour (dose given are in µg/kg·min).

To calculate the value of CRP and SOFA score in clinical decision making, we defined the following interventions as events: CT-scan, ultrasonography (with or without punction), and flexible endoscopy or (re) laparotomy/thoracotomy. When surgical complications were found during these interventions, the events were defined as positive. Surgical complications were defined as intra-abdominal abscesses, anastomotic leakage, mesenterial ischemia, ileus, perforations, bleeding, diaphragm rupture or pneumothorax. Other non-surgical complications were also registered if present.

The treating physicians were not blinded for CRP nor SOFA score in order to reflect actual practice on the ICU. In addition, the majority of the treating physicians was not involved, nor participated in the design, purpose and methods of the study.

The number of historical ICU-episodes to be retrieved in this observational study is determined by the smallest group: the number of complication as proportion of the number of events to be predicted by two consecutive CRP measurement and SOFA measurements. Based on the rule of thumb that in logistic regression analysis the size of the smaller group should be at least ten times the number of variables in the model, the number of historical ICU episodes to be retrieved and the number of events should be large enough to yield at least 40 complications. We anticipated that 40 complications would result from 100 first events which in its turn would result from 200 ICU-episodes.

### Statistical Analysis

Results are presented as means ± standard deviation for continuous variables that have a Gaussian-shaped distribution and as (relative) frequencies for categorical nominal variables. When comparing two groups, the Chi-square test was used for categorical nominal variables and the unpaired T-test for continuous variables.

The relationship between the observation that an event had taken place and the preceding levels or changes of CRP was analyzed in a case-cohort design using conditional logistic regression analysis. Time was measured as days since admission to the ICU. For each day that an event occurred, the last and next to last CRP measurements were compared between patients with an event (the cases) and patients without an event on that same day (the remaining cohort). This comparison was made by using conditional logistic regression analysis. In this analysis the data were stratified by event-day, providing an optimal adjustment for day since admission when estimating the simultaneous effect of level and change of CRP on the event probability. Only the first occurring event within a patient was involved in this analysis so that events can be considered as mutual independent outcomes.

CRP-measurements were logarithmically transformed before analysis because of their positive skewness. The difference and sum of both measurements were entered in the model, respectively standing for the simultaneous effect of CRP-change and CRP-level on the event probability.

Directly after an event had taken place in a patient, additional investigations were done to confirm the event. So, in all ICU-patients in whom a first event had taken place a second outcome variable was observed: confirmation (yes or no). That outcome variable was analyzed using ordinary logistic regression analysis with the same explanatory CRP-variables as in the conditional regression analysis for explaining event (yes/no).

Prior to the above described (conditional) logistic regression analyses we analyzed the dependency of CRP on ICU-day (a maximum of 28 days) and event (yes/no) using linear mixed modelling. The first aim was to estimate the mean difference in CRP between events and no events across all 28 days (at maximum) and across all subjects in the total ICU-cohort, while correcting for the categorical day-effect on CRP. In this analysis all events within a patient were taken into account by considering event (yes/no) a time-dependent within-patient explanatory factor equal to 1 at ICU-days that an event occurred and 0 at other ICU-days. The variance-covariance structure of the repeated CRP measurements across ICU-days was assumed to be first-order autoregressive with heterogeneous variances. A similar analysis was done for the effect on complication (yes/no). The second aim was to analyze the dependency of CRP levels on the SOFA score entered in the linear mixed model.

Statistical analysis was performed using SPSS 15.0, GraphPad Prism 5.0 for Windows and EGRET. A two sided *P*-value below 0.05 was considered to indicate statistical significance.

## Results

A total 174 patients were included in the study with 198 ICU-episodes. Four patients had three ICU-episodes, 16 patients had two ICU-episodes and 154 had one ICU episode. At time of admission into the ICU, the mean age of our patient population was 72 years (±12 years, minimum 26 years and maximum 91 years). Postoperative SIRS and sepsis were the main indications for admission in 45% (n = 78) of all patients. Most patients admitted into the ICU had undergone an emergency laparotomy for an acute abdomen or a re-exploration for complication of previous surgery (18% and 14%, respectively). Further baseline characteristics between patients with and without events are showed in table 2. The groups were not overall similar, for example the operations before admission into the ICU and the APACHE II score differed between groups (Table 2).

**Table 2:** Patients characteristics of patients with an event versus patients without an event (N = 174).

	No event (N = 97)	Event (N = 77)^1^	P-value
Age *mean (SD)*	71.0 (12.3)	72.2 (11.1)	0.51
Male *N (%)*	61 (62.9)	53 (68.8)	0.41
Length of Stay on ICU *mean (±SD)*	5.0 (4.3)	9.9 (6.7)	<0.0005
Apache II score *mean (±SD)*	19.1 (6.5)	22.5 (8.0)	0.003
*Indication for admission into ICU*			0.40
Standard postoperative care after elective major surgery *N (%)*	16 (16.5)	9 (11.7)	
Postoperative with SIRS/sepsis *N (%)*	40 (41.2)	38 (49.4)	
Respiratory insufficiency *N (%)*	21 (21.6)	13 (16.9)	
Vascular operation *N (%)*	5 (5.2)	1 (1.3)	
Primary sepsis/AKI/MOF *N (%)*	8 (8.2)	11 (14.3)	
Others^2^ *N (%)*	7 (7.2)	5 (6.5)	
*Indication for operation before admission into ICU*			0.047
No operation *N (%)*	15 (15.5)	16 (20.8)	
Elective colorectal operation *N (%)*	8 (8.2)	7 (9.1)	
Ileus *N (%)*	6 (6.2)	4 (5.2)	
Acute abdomen *N (%)*	21 (21.6)	11 (14.3)	
Epigastric region *N (%)*	6 (6.2)	2 (2.6)	
Complication of prior operation *N (%)*	10 (10.3)	14 (18.2)	
Embolectomy, bypass, amputation *N (%)*	8 (8.2)	1 (1.3)	
Elective correction AAA *N (%)*	3 (3.1)	12 (15.6)	
Ruptured AAA *N (%)*	9 (9.3)	5 (6.5)	
VATS or thoracotomy *N (%)*	2 (2.1)	1 (1.3)	
Others^3^ *N (%)*	9 (9.3)	4 (5.2)	
Mortality *N (%)*	6 (6.2)	11 (14.3)	0.074

N = number SD = standard deviation % = percentage of total patients events or no events.

1 The number of patients who had an event was lower than the total number of events.

2 Others indication for admission are cardiovascular observation without prior operation.

3 Others indication for operation are coiling, surgery after trauma or stabilization of fracture.

SIRS = systemic inflammatory response syndrome.

AKI = acute kidney injury.

MOF = multi organ failure.

Acute abdomen = includes perforations, mesenterial ischemia, peritonitis.

Epigastric region = Whipple, cholecystectomy, BII stomach resection, splenectomy.

Complications = mesenterial ischemia, anastomotic leakage, platzbauch, intra-abdominal abscess.

AAA = aneurysm of the abdominal aorta.

The mean Acute Physiology and Chronic Health Evaluation II score on admission was 21±9 points (min 8 - max 49 points). Patients with an event had significant higher scores than those without an event (23±8 points versus 19±7 points, respectively; *p* = 0.003). No significant difference in APACHE II score was seen between patients with and without complications (22±8 and 24±8, respectively; *p* = 0.22).

Overall in-hospital mortality on the ICU in our patient group was 9.8% (17/174). A higher mortality rate was observed in patients who underwent an event compared to those without an event (14.3% versus 6.2%, *p* = 0.07). Patients with and without complications had a small, non-significant difference in mortality (15% versus 13%, respectively; *p* = 1.00). The main cause of death was multi organ failure.

**Figure 1:**
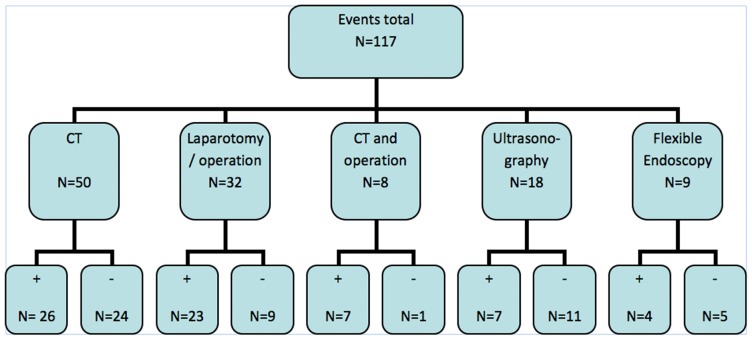
Events and outcome. Illustration of event type and its outcome, complication or not. N = number+ = actual complication found − = no complication found.

Seventy-seven patients (47%) of the 174 patients admitted into the ICU had an event of which twenty-four underwent more than one intervention. A total of 117 events were identified of which 50 (43%) were CT-scans in 30 patients and 32 (27%) re-operations in 23 patients (Table 3). In 48% of the CT-scans made, no complications were found. In 61% of the ultrasonograms, no complications were found (Figure 1). If the event was an operation and the decision to perform surgery was based on the clinical parameters without radiological findings, in 72% of the cases an actual complication was found.

**Table 3:** Description of event type.

	Patients (N = 174)	Total events (N = 117)
No events *N (%)*	97 (55.7)	–
CT-scan *N (%)*	30 (17.2)	50 (42.7)
Laparotomy or operation*N (%)*	23 (13.2)	32 (27.4)
CT and operation *N (%)*	7 (4.0)	8 (6.8)
Ultrasonography *N (%)*	13 (7.5)	18 (15.4)
Flexible endoscopy *N (%)*	4 (2.3)	9 (7.7)

% = percentage of total N = number.

**Figure 2:**
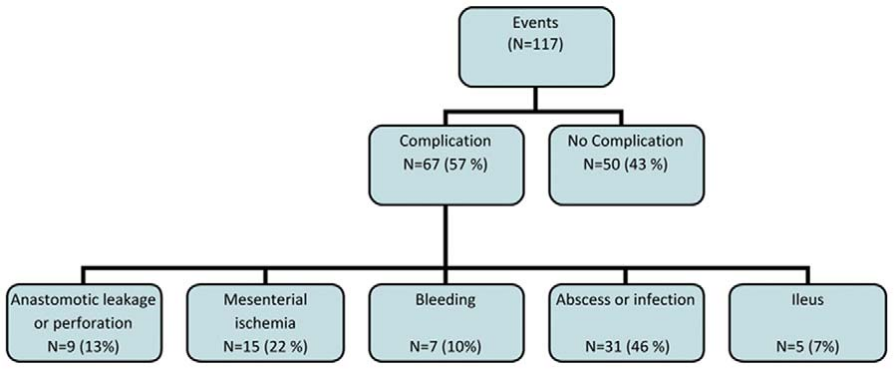
Event and complications. Description of complication type when found. N = number % = percentage of events/complications.

One-hundred and seventeen events occurred in 77 of the 174 patients admitted into the ICU. Of these 117 events, 67 (46/77 patients) were involved with a complication. The most common complications that we found were intra-abdominal abscesses (n = 31; 46%) followed by mesenterial ischemia (n = 15; 22%) (Figure 2). Non-surgical infectious complications found in this population were mainly pneumonias. An overview of non-surgical complications are shown in table 4.

**Table 4:** Description of non-surgical complications.

	No event (N = 67)	Event (N = 77)
Pneumonia *N (%)*	22 (12.6)	18 (10.3)
Sepsis with infected central venous lines *N (%)*	3(1.7)	4 (2.3)
Wound infection *N (%)*	1 (0.5)	0 (0)
Positive cultures for ascites *N (%)*	0 (0)	1 (0.5)

% = percentage of total N = number.

The Sequential Organ Failure Assessment Score varied between 0 and 16 points. At admission a mean SOFA score of 5.2±2.6 points was found, which peaked at day two (5.6±2.7 points). After this peak, the mean SOFA score decreased over time. No significantly different SOFA score was observed between patients with and without an event (mean difference −0.10 points; 95% CI: −0.32 to +0.13; *p* = 0.39). Furthermore, patients with and without complications had no difference in SOFA scores either (−0.70 points; 95% CI: −1.58 to +0.19; *p* = 0.12). On the other hand, a significant log-linear relationship between SOFA score and CRP concentrations was found using linear mixes modeling. A one point increase in SOFA score corresponded with a 7.2% increase in CRP levels (95% CI: 5.3%–9.0% *p*<0.0005).

**Figure 3:**
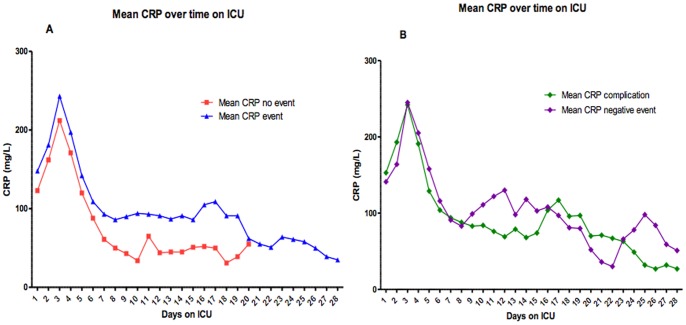
Mean C-reactive protein concentrations over time. A Mean C-reactive protein concentrations over time in surgical patients in the ICU with and without events. **B** Mean C-reactive protein concentrations of surgical patients with negative events and positive events (complications).

The overall mean CRP levels of patients included in the study peaked on day 3 of admission (mean 226±114 mg/L). In patients with and without an event, CRP concentrations also peaked on day 3 (243±116 mg/L and 212±111 mg/L, respectively; *p* = 0.080). After this peak the CRP concentrations started to decrease to normal levels (Figure 3). CRP concentrations in patients with an event showed a 7.5% higher CRP level compared to patients without an event on the same day (95% CI 0.3%–15.2%; *p* = 0.040). No significant difference in CRP levels was seen when comparing patients with a complications and without a complication on the same day (−4.7%; 95% CI: −26.8%to +24.7%; *p* = 0.73). In these analyses linear mixed modeling accounted for all available daily repeated CRP measurements within patients during the ICU stay.

All 117 events contributed to the linear mixed model analyses, whereas in the (conditional) logistic regression analyses only the 77 first events were included. In the conditional logistic regression analysis we stratified on 10 different event days, with the number of events (cases) decreasing from 27 to 4 with increasing event day and the number of patients in the remaining event-free cohort dropping off from 137 to 7 due to ending of ICU episodes. Meanwhile relatively small numbers of missings arose caused by incidentally missing last CRP changes or last SOFA score changes just before the event-day considered.

The effect on the odds of an event resulting from a relative increase in percent change of CRP, adjusting for ICU day and CRP level (and additionally for change and level of SOFA score) was estimated with the conditional logistic regression analysis. Effects of additive changes in SOFA score were estimated as SOFA score was not transformed in the analysis. Concerning CRP, effects of multiplicative (relative) changes were studied, as CRP was logarithmically transformed before analysis. From the analysis we found that each additional 10% increase in percent change of CRP resulted in a 3.5% increase in the odds of a first event (OR 1.035; 95% CI: 1.004–1.068, *p* = 0.028). A 10% increase in CRP level resulted in a 2.6% increase in the odds for an event (OR 1.026; 95% CI: 0.997–1.055; *p* = 0.075). The p-value of the simultaneous effect of CRP change and level on the event odds is 0.059 (Table 5, Model 1).

**Table 5:** Effects of CRP and SOFA on the odds of an event or complications.

Model	Outcome events		Outcome complications	
	OR (95% CI)	*p*	OR (95% CI)	*p*
model 1: CRP		0.059		0.39
10% increase of change factor	1.035 (1.004–1.068)	0.028	0.983 (0.932–1.036)	0.52
10% increase of level	1.026 (0.997–1.055)	0.075	1.009 (0.973–1.066)	0.44
model 2: SOFA		0.18		0.60
1 point larger increment	1.055 (0.925–1.204)	0.42	1.094 (0.876–1.366)	0.43
1 point larger level	1.073 (0.986–1.168)	0.10	0.932 (0.779–1.117)	0.45
model 3: CRP+SOFA		0.12		0.49
10% increase of change factor in CRP	1.033 (1.001–1.065)	0.046	0.980 (0.929–1.034)	0.46
10% increase of CRP level	1.022 (0.994–1.052)	0.13	1.021 (0.929–1.034)	0.40
1 point larger increment in SOFA score	1.037 (0.908–1.185)	0.59	1.156 (0.914–1.462)	0.23
1 point larger level of SOFA score	1.055 (0.966–1.151)	0.23	0.965 (0.799–1.164)	0.71

A one point increase in SOFA score and an additional one point increase in SOFA score resulted respectively in a 5.5% higher event odds (OR 1.055; 95% CI: 0.925–1.204; *p* = 0.42) and a 7.3 higher event odds (OR 1.073; 95% CI: 0.986–1.168; *p* = 0.10). The p-value of the simultaneous effect of SOFA change and level on the event odds was 0.18 (Table 5, Model 2). The effects of changes and levels of both CRP and SOFA scores simultaneously, and hence adjusted for one another, are presented as model 3 in Table 5. The overall p-value of those four effects is 0.12.

Using the data of the 77 first events, of which in 46 events a complication was confirmed, an ordinary logistic regression analysis was done in order to try to explain the probability of a complication from preceding CRP changes similar to the conditional logistic regression analysis described above. No significant effect of CRP change on the probability of a complication was found. Each additional 10% increase in percent change of CRP resulted in a 1.7% decrease in the odds of a complication (OR 0.983; 95% CI: 0.932–1.036, *p* = 0.52). Again after adjusting for SOFA a similar non-significant result was obtained (OR 0.980; 95% CI: 0.929–1.034; *p* = 0.46) (Table 5). It can be concluded that the only significant effect found is that of CRP changes on the probability of an event. The decision of the treating clinicians to do an intervention is apparently partly based on the last observed CRP change.

## Discussion

To determine if a diagnostic and/or therapeutic (re)interventions are indicated in the critically ill surgical patient during the ICU stay, trends in CRP concentrations are often used. In this large prospective study with critically ill general surgical patients we found a significant relation between increase in CRP concentrations and events. However, no significant relation between increase in CRP levels and complications was found.

Although many studies showed that increasing CRP concentrations or persistently high CRP levels are suggestive for ongoing inflammation with multi organ failure and poor outcome, these studies mainly included a general ICU population [5], [6], [12]. In our study we evaluated the CRP levels and events of only critically ill surgical patients in the ICU.

C-reactive protein has been considered as an indicator for adverse postoperative course including both surgical and non-surgical complications as it responds to both infection and inflammation. Mustard et. al. [12] found in 1987 that a normal CRP response to surgery without a secondary rise can be used to exclude the possibility of a postoperative septic complication, due to its negative predictive value of 78%. In spite of the fact that this study included a small surgical population (n = 108), they concluded that CRP measurements should be used as adjunct to surgical care in patients at high risk for postoperative complications [12]. However, a negative predictive value of 78% is low. Further information on these complications is available from previous studies examining complications after colorectal surgery. Intra-abdominal infection caused by an anastomotic leak after colorectal surgery was correlated with prolonged CRP concentrations over 125–190 mg/L or higher on the third postoperative day [10]. Similarly, this persistent CRP elevation and levels higher than 140 mg/L on postoperative [9], [13], [14], [15] days 3 and 4 were predictive for infectious postoperative complications (86% and 91%) [9]. Despite of our large group of patients, we could not confirm this finding. In our study population, a value >140 mg/L was not related to complications in the conditional logistic analysis. Yet, trends in CRP levels during the first 48 hours of ICU admission can be helpful in the decision whether or not further diagnostic procedures are needed in critically ill patients [5]. Our study showed a significant relation between 10% to 30% increase in CRP concentrations and events (diagnostic or therapeutic procedures). However, this increase in CRP levels showed no significant relation with surgical complications. Platt et.al. also confirmed higher CRP levels in patients with postoperative infectious complications compared to those without any complications [16]. Nevertheless, this large study could not differentiate between patients with surgical and non-surgical infectious complications as well.

Since it is difficult to diagnose early complications in critically ill patients, other parameters may also be used in the decision making for further diagnostic or therapeutic procedures. These may include the Sequential Organ Failure Assessment Score (SOFA) by means of clinical deterioration. The usefulness of SOFA score in critically ill patient has been validated in large cohorts demonstrating its prediction for mortality and outcome [17], [18]. With regards to our findings, an increase in mean SOFA score during the first 48–72 hours of admission was often seen. Nevertheless, no difference in SOFA score between patients with and without events and with or without complications was found during these 72 hours or rest of the stay in the ICU. Even when CRP was adjusted for the SOFA score, only a significant relation with events was found, but none with complications.

It appears that an increase in CRP levels triggers physicians to perform additional interventions (events). As no relationship between an increase in CRP and postoperative complications is found, we can argue if CRP is a reliable marker. It is important to realize that changes in CRP concentrations should not be interpreted separately from clinical data in clinical decision making. Newer markers are needed and currently being studied as parameters for infection and sepsis and other complications.

There are several limitations to our study. The first is that the treating physicians were not blinded for CRP or the SOFA score. We realize that this is a risk for bias. The reason for not blinding the clinician is that we wanted to reflect practice as it is on the ICU. As the clinicians were not blinded for CRP concentrations or SOFA scores, it may have prompted the physicians to order diagnostic tests or perform interventions based on changes in CRP levels or SOFA scores. Yet, this is a reflection of actual practice in the ICU and clinicians should not make decisions based on isolated laboratory values or SOFA scores alone. Another downside of our study is that also non-infectious complications (surgical or non-surgical) were included in the analysis. For example patients with postoperative bleeding, ileus or mestenterial ischemia. These patients developed a complication which was not necessary accompanied by an increase in CRP. As this was only a small amount of patients, we can assume that they did not influence our data. Furthermore, as mentioned above our data provided us with more reliable information on the clinical course of the general surgical patients on the ICU. In addition, it might be that in different hospital region or countries, the threshold to perform a diagnostic or therapeutic intervention may differ from our hospital. Nevertheless, the patients included in our study were those admitted into a large level 3 ICU with a broad surgical population and different physicians. We therefore believe that this study is representative for a general critically ill surgical patients admitted into an ICU.

### Conclusions

In this observational study we conclude that increase in C-reactive protein concentration is a poor parameter for detection of surgical complications in the critically ill surgical patient in the ICU by means of diagnostic procedures or therapeutic (re-)interventions. Yet, we found that when CRP increased, more diagnostic and therapeutic interventions were performed. In addition, we recommend that CRP should not be used by itself for clinical decision making but in adjunct to clinical parameters. Since a combination with the SOFA score does not seem to improve its value, more reliable clinical scoring systems and markers are needed.
